# Case report: Successful bronchoscopic interventional treatment of endobronchial leiomyomas

**DOI:** 10.1515/biol-2022-0845

**Published:** 2024-05-07

**Authors:** Yinfeng Wang, Yixiang Zhang, Ruirui Tong

**Affiliations:** Department of Pulmonary and Critical Care Medicine, Second Affiliated Hospital of Fujian Medical University, Respiratory Medicine Center of Fujian Province, No. 34, North Zhongshan Road, Licheng District, Quanzhou 362000, Fujian, China; Department of Pathology, Second Affiliated Hospital of Fujian Medical University, Quanzhou 362000, Fujian, China

**Keywords:** endobronchial leiomyomas, minimally invasive intervention, bronchoscopy, pulmonary leiomyoma, case report

## Abstract

Endobronchial leiomyomas are rare benign neoplasms of the lungs that arise from the smooth muscle cells of the bronchi and bronchioles. While surgical resection is the mainstay of treatment for these tumors, bronchoscopic interventional therapies are also effective and can help preserve lung function in certain cases. A 40-year-old male patient presented with a persistent cough and sputum production for over 4 months. A chest computed tomography scan revealed nodular lesions in the lower lobe bronchus, later confirmed as an endobronchial leiomyoma. The patient refused surgical intervention and opted for minimally invasive bronchoscopic treatments, including electric snare resection, argon plasma coagulation, and balloon dilation, resulting in a successful outcome with no recurrence during follow-up. Clinicians should consider bronchoscopic interventions as a viable treatment option for endobronchial leiomyomas patients who are either ineligible for surgical resection or opt not to undergo surgery.

## 1 Introduction

Endobronchial leiomyomas are rare benign tumors originating from the smooth muscle cells of the bronchial tree [1,2]. Although uncommon, these tumors can cause significant respiratory symptoms and pose a diagnostic and therapeutic challenge. Traditional treatment options for endobronchial leiomyomas have involved surgical resection, which carries the risk of complications and prolonged hospital stays [3]. In recent years, bronchoscopic intervention techniques have emerged as a minimally invasive alternative for the management of endobronchial tumors, including leiomyomas [4].

The advent of advanced bronchoscopic technologies, such as flexible bronchoscopy and interventional tools, has revolutionized the field of interventional pulmonology [5]. These techniques allow for precise visualization, biopsy, and therapeutic interventions within the bronchial tree, offering a less invasive approach with reduced morbidity and faster recovery times [6]. With the increasing use of bronchoscopic intervention, its application in the treatment of endobronchial leiomyomas has gained attention [7]. However, the optimal treatment strategy for these rare tumors remains controversial, and there is limited literature available on the management of these tumors using bronchoscopic interventions. In this case report, we present a successful case of endobronchial leiomyoma treated solely through bronchoscopic intervention.

## 2 Case presentation

A 40-year-old male patient presented to our department on May 16, 2022, with a chief complaint of persistent cough for over 4 months. The cough was accompanied by productive sputum, which was white and of significant quantity. The patient denied experiencing fever, chest tightness, dyspnea, or hemoptysis. Initial treatment at a local clinic did not provide substantial relief, and the cough and sputum production persisted. The patient developed wheezing 2 months before admission and was subsequently referred to our hospital for further evaluation. The patient had a history of chronic gastritis for more than ten years. There were no significant personal, obstetric, or familial medical histories. Upon admission, the patient’s vital signs were within normal ranges. Physical examination revealed no abnormalities in the skin, mucous membranes, superficial lymph nodes, lungs, heart, abdomen, or extremities.

**Figure 1:**
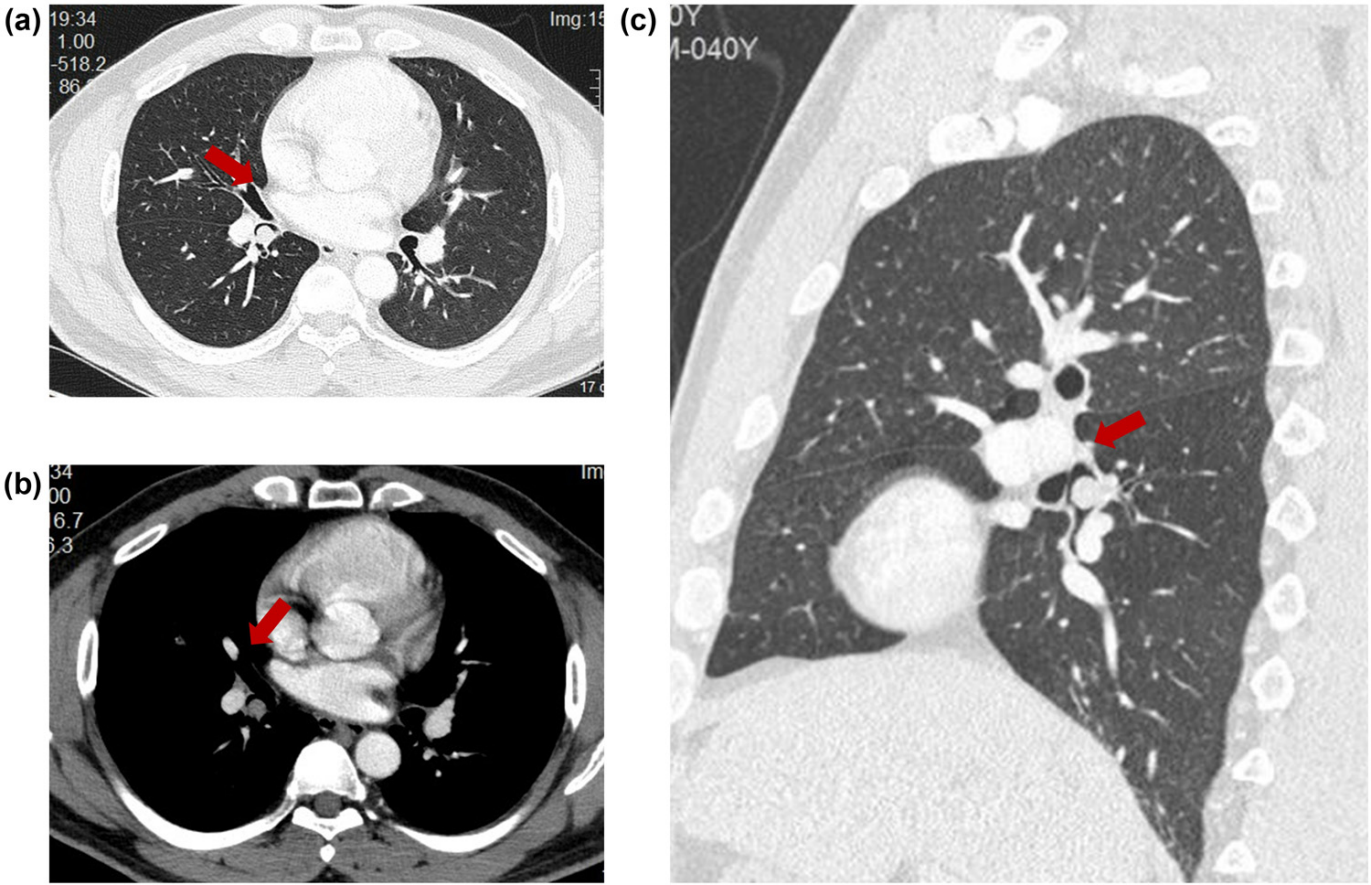
Chest CT scan with or without contrast. (a and b) Small nodules in the bronchial lumen of the dorsal segment of the lower lobe of the right lung. (c) Scattered tiny nodules in both lungs.

To further investigate the patient’s condition, a chest computed tomography (CT) scan was performed, which revealed the following findings: (1) a small nodular lesion within the right lower lobe bronchus and (2) scattered small nodules throughout both lungs. Given these findings, the patient was admitted to our department with a preliminary diagnosis of “occupying lesion of the lung” (Figure 1).

**Figure 2:**
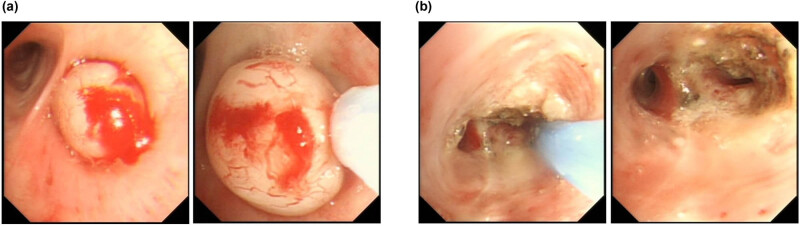
Bronchoscopic appearance (a) before and (b) after minimally invasive intervention.

Upon admission, the patient underwent bronchoscopy for a comprehensive evaluation. During bronchoscopy, a white oval-shaped lesion completely obstructing the right lower lobe bronchus was observed (Figure 2a). The lesion exhibited some mobility, and the bronchoscope could pass through a narrow gap distally. The lesion was treated using electrocautery and retrieved with a basket forceps. Subsequently, residual tumor tissue at the opening of the right lower lobe bronchus was treated with electrocautery and a ball electrode (Figure 2b), followed by mucosal biopsy.

**Figure 3:**
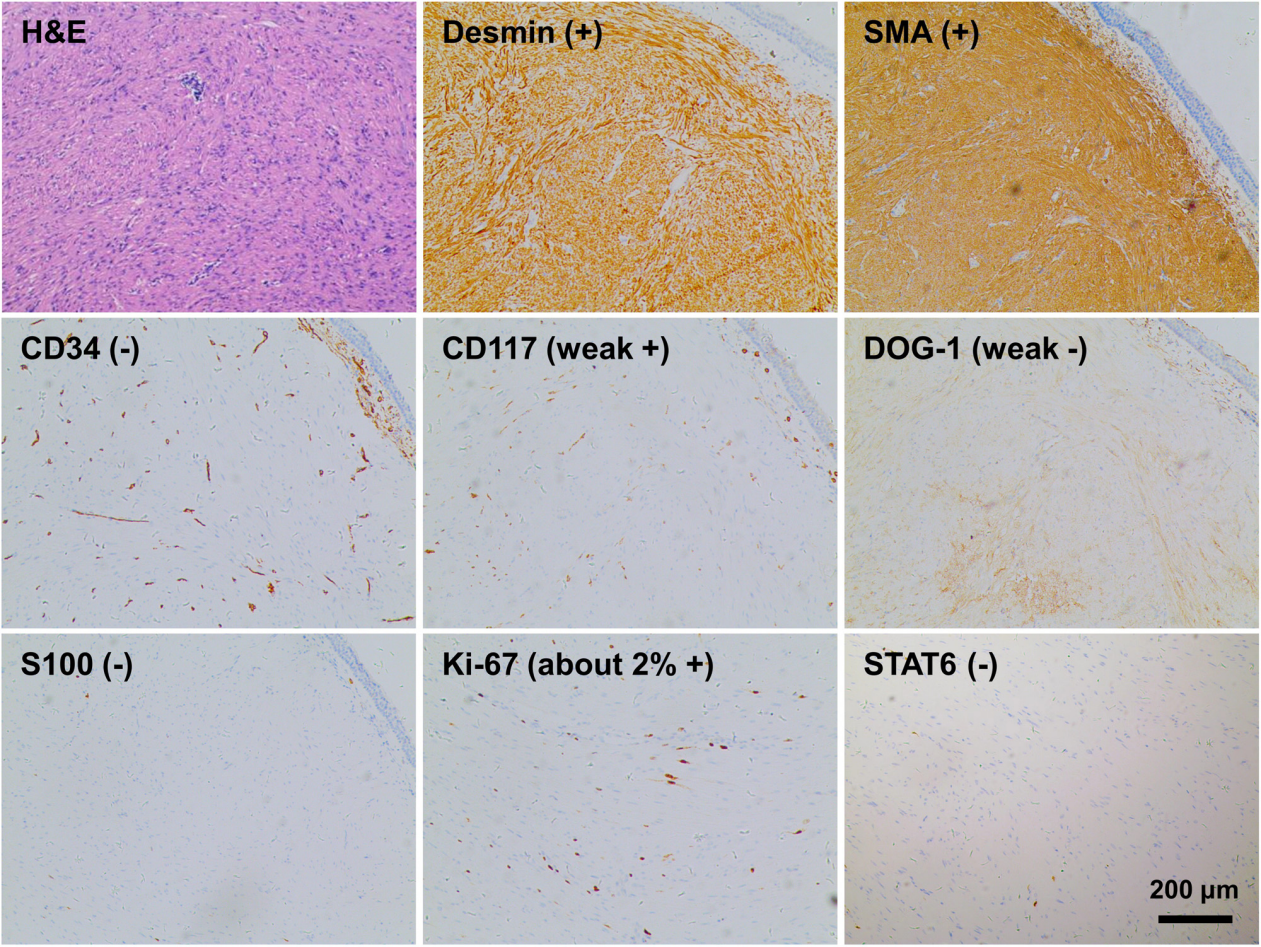
Diagnostic tumor pathology (magnification: 10×).

Histopathological examination of the specimen obtained during bronchoscopy revealed a spindle cell tumor with bundled arrangement and inconspicuous atypia, consistent with leiomyomas (Figure 3). Immunohistochemical analysis demonstrated Desmin (+), SMA (+), CD34 (−), CD117 (weak +), DOG-1 (weak −), S100 (−), Ki-67 (about 2% +), and STAT6 (−). These findings confirmed the diagnosis of endobronchial leiomyomas.

**Figure 4:**
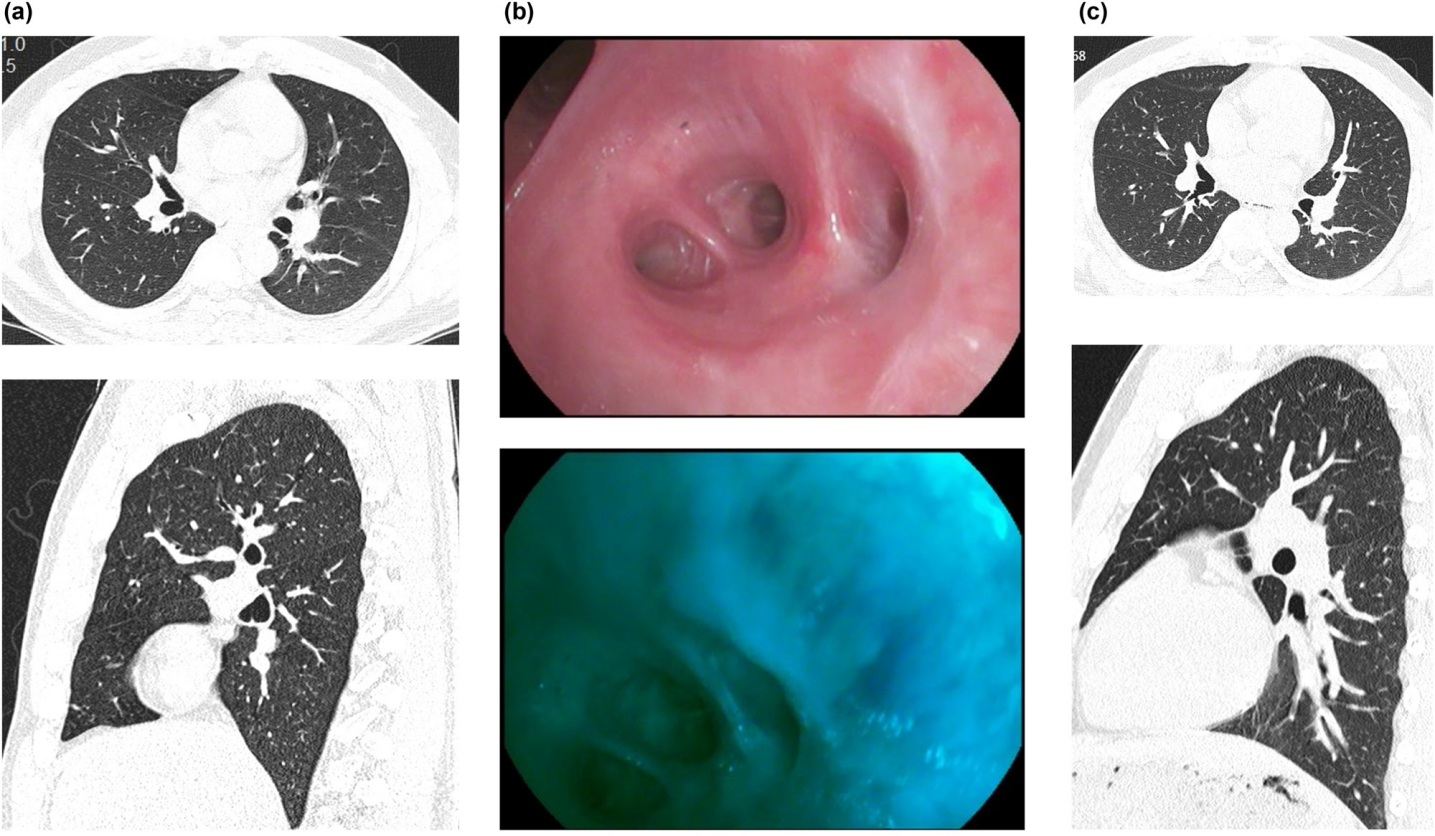
Postoperative follow-up review. (a) A chest CT scan at 3 weeks postoperatively showed a soft tissue nodule in the right bronchus, but there was no significant change compared to the previous examination. (b) Bronchoscopy performed at 7 weeks postoperatively showed localized mucosal whitening and fluorescence defects at the right lower lobe bronchial opening, but the airway remained patent. (c) A chest CT scan 6 months postoperatively showed no signs of tumor recurrence.

Based on the confirmed diagnosis, surgical intervention was recommended to the patient. However, the patient and his family declined surgery. Consequently, a repeat bronchoscopic intervention was performed to address any residual or recurrent tumor. The necrotic tissue was cleared, and argon plasma coagulation was applied to the affected area in the posterior segment of the right lower lobe. Following the procedure, the patient showed improvement and was discharged. Subsequent follow-up examinations, (Figure 4) including chest CT and bronchoscopy, did not reveal any signs of tumor recurrence.

At the 3-week postoperative visit, a chest CT scan demonstrated a soft tissue nodule within the right bronchus, but it showed no significant changes compared to the previous examination. A bronchoscopy performed 7 weeks postoperatively showed localized mucosal whitening and fluorescence defects at the opening of the right lower lobe bronchus, but the airway remained patent. No evidence of tumor recurrence was observed. At the 6-month postoperative follow-up, a chest CT scan showed no signs of tumor recurrence, providing further evidence of successful treatment.


**Informed consent:** Informed consent has been obtained from all individuals included in this study.
**Ethical approval:** The research related to human use has been complied with all the relevant national regulations, institutional policies and in accordance with the tenets of the Helsinki Declaration, and has been approved by the Medical Ethics Committee of Second Affiliated Hospital of Fujian Medical University (Approval Number 2023-242).

## 3 Patient perspective

The patient in this case report had concerns about undergoing surgery, which led him to explore alternative treatment options. He and his family made an informed decision to pursue bronchoscopic interventions, considering the potential benefits and risks. The patient’s willingness to actively participate in the decision-making process and his commitment to follow-up examinations contributed to the successful outcome of the treatment. Patient-centered care, which includes considering the patient’s preferences and values, played a crucial role in tailoring the treatment approach to meet the individual needs of the patient.

## 4 Discussion

The successful treatment of endobronchial leiomyomas with bronchoscopic intervention presents a promising alternative to traditional surgical resection. In this case report, we demonstrated the feasibility and efficacy of bronchoscopic techniques in the management of endobronchial leiomyomas, a rare tumor, highlighting the advantages of this minimally invasive approach. Bronchoscopic intervention provides several key benefits in the treatment of endobronchial leiomyomas. It allows for direct visualization of the tumor within the bronchial tree, facilitating accurate diagnosis and localization [8]. Flexible bronchoscopy, with its ability to maneuver through the bronchial tree and access peripheral airways, enables precise identification of the tumor’s location and extent. This information is crucial for planning and executing the therapeutic intervention. Surgical removal is the traditional method of treating endobronchial leiomyomas. Depending on the size and location of the tumor, as well as the extent to which the distal bronchus affects the lungs, the surgeon may choose a different surgical approach. For smaller localized tumors, local excision of the tumor may be used, while in cases where the tumor is located at the bronchus, bronchial circumferential resection and end-to-end anastomosis at the tumor site may be necessary [9,10]. If the tumor leads to bronchial obstruction, obstructive pneumonia, lung solidity, or atelectasis, it may be necessary to remove the corresponding lung lobe at the same time, or even perform a one-sided lung resection. Over traditional surgical approaches, minimally invasive bronchoscopic interventions eliminate the need for invasive surgery, reducing the associated risks, such as postoperative complications, prolonged hospital stays, and recovery time. Furthermore, the preservation of lung parenchyma and bronchial integrity with bronchoscopic techniques contributes to better long-term pulmonary function outcomes [11]. These interventions include electrocautery, laser therapy, cryotherapy, and argon plasma coagulation, among others [12]. The success of bronchoscopic intervention is highly dependent on the size, location, and accessibility of the tumor [13]. The presence of endobronchial obstruction or significant airway stenosis may necessitate the use of adjunctive therapies, such as airway stenting, to maintain airway patency. In this case, electrocautery and argon plasma coagulation were employed, effectively treating the endobronchial leiomyomas.

The main symptom in our case was a persistent cough accompanied by productive sputum for more than 4 months. This is consistent with previous reports of endobronchial leiomyomas, where cough and sputum production are commonly observed [14]. However, it is important to note that some patients may also present with other symptoms such as dyspnea, hemoptysis, or chest pain, which were absent in our case [15]. The patient’s medical history was notable for a 10-year history of chronic gastritis, and there were no remarkable personal, reproductive, or family history. In the process of diagnosing the presented case of endobronchial leiomyomas, alternative perspectives and potential differential diagnoses were considered to ensure a comprehensive evaluation. One potential alternative diagnosis that may present with similar symptoms is bronchial asthma. Asthma is characterized by recurrent episodes of cough, wheezing, and shortness of breath [16]. In this case, the patient did report symptoms of cough and subsequent development of wheezing. However, there were several aspects of the patient’s clinical presentation and diagnostic findings that did not align with asthma. The absence of fever, chest tightness, or significant dyspnea made the diagnosis of asthma less likely. Another potential consideration in this case could be infectious bronchitis or pneumonia. The presence of a persistent cough with productive sputum might raise suspicion of an infectious etiology. However, the lack of accompanying symptoms such as fever, chest pain, or systemic signs of infection made the possibility of infectious bronchitis or pneumonia less likely. Considering the patient’s medical history of chronic gastritis, another potential explanation for the symptoms could be gastroesophageal reflux disease (GERD). Chronic cough can be a manifestation of GERD, especially if there is associated regurgitation of gastric contents [17]. However, in this case, there are no typical symptoms of GERD, such as heartburn or acid regurgitation [17]. Additionally, the chest CT scan reveals nodular lesions within the bronchi, and the bronchoscopy examination identifies an obstructive tumor within the bronchus, providing a clear cause for the patient’s symptoms. These factors collectively contribute to the exclusion of alternative diagnoses such as asthma, infectious bronchitis or pneumonia, and GERD.

Histopathological examination is essential for confirming the diagnosis of endobronchial leiomyomas. Immunohistochemical analysis aids in distinguishing this tumor from other neoplasms. Positive staining for smooth muscle markers, such as Desmin and SMA, supports the diagnosis, while negative staining for CD34 and S100 helps rule out other possibilities [18]. In this case, the histopathological examination revealed spindle-shaped cellular arrangements observed in the hematoxylin and eosin staining, along with positive immunohistochemical staining for Desmin and SMA. These findings confirm the neoplasm’s smooth muscle origin. The Ki67 proliferation index was low (approximately 2%), indicating a low mitotic activity. These findings are consistent with the diagnosis of a benign endobronchial leiomyoma. The optimal treatment for pulmonary leiomyomas is surgical resection. This approach aims to achieve complete tumor removal and minimize the risk of recurrence. However, in our case, the patient and his family refused surgical intervention. Instead, bronchoscopic intervention was performed, including electrocautery snaring, basket forceps extraction, and subsequent argon plasma coagulation for residual tumor treatment. This alternative management strategy was successful in our case, as evidenced by the absence of tumor recurrence during follow-up. The decision to opt for bronchoscopic intervention reflects the importance of individualized patient care and the consideration of patient preferences and comorbidities. Long-term follow-up is crucial for evaluating the effectiveness and durability of bronchoscopic intervention in the treatment of endobronchial leiomyomas. Close monitoring of tumor recurrence and the occurrence of complications is necessary to ensure the long-term success of the procedure. It is important to consider the potential for late tumor recurrence or regrowth, as well as the possibility of secondary malignancies developing in the bronchial tree. Regular bronchoscopic assessments and imaging studies are recommended for ongoing surveillance, aiming to detect and manage any recurrence or new lesions.

This case highlights the importance of considering endobronchial leiomyomas as a potential differential diagnosis in patients presenting with persistent cough and sputum production. Although these tumors are rare, they should be considered in the evaluation of patients with obstructive symptoms and bronchial lesions. This case also emphasizes the value of bronchoscopic interventions as an alternative management strategy when surgical resection is not feasible or preferred. The successful outcome in this case suggests that bronchoscopic interventions, such as electrocautery snaring and argon plasma coagulation, can be effective in treating endobronchial leiomyomas and preserving lung function. Clinicians should be aware of this treatment option, particularly for some patients who are too old, have multiple underlying diseases and have poor cardiopulmonary function to tolerate surgical resection. While this case report provides valuable insights, it is essential to acknowledge its limitations. First, this report is based on a single case, which inherently restricts the generalizability of the research findings. Another potential limitation is the relatively short follow-up period, as the absence of tumor recurrence needs to be validated over a longer period of time to confirm the efficacy of bronchoscopic intervention. Further studies comparing the outcomes of surgical resection and bronchoscopic intervention in a larger patient population would contribute to elucidating the optimal treatment approach. Additionally, further research is needed to explore the genetic and molecular characteristics of these tumors, which could provide information for targeted therapies or prognostic markers.

## 5 Conclusion

This case report highlights the successful management of an endobronchial leiomyoma using electrocautery and argon plasma coagulation during bronchoscopy. The patient experienced symptom relief, and no tumor recurrence was observed during the follow-up period. Minimally invasive bronchoscopic interventions provide an effective means of treating benign lung tumors while preserving lung function and enhancing the patient’s quality of life, particularly in patients who are not suitable candidates for surgery or decline surgical treatment. Further research and more extensive studies are warranted to establish the efficacy and long-term outcomes of bronchoscopic interventions in the management of endobronchial leiomyomas.
